# Cerebellar volumetry in ataxias: Relation to ataxia severity and duration

**DOI:** 10.21203/rs.3.rs-3605029/v1

**Published:** 2023-11-16

**Authors:** Mónica Ferreira, Tamara Schaprian, David Kügler, Martin Reuter, Katharina Deike-Hoffmann, Dagmar Timmann, Thomas M. Ernst, Paola Giunti, Hector Garcia-Moreno, Bart van de Warrenburg, Judith van Gaalen, Jeroen de Vries, Heike Jacobi, Katharina Marie Steiner, Gülin Öz, James M. Joers, Chiadi Onyike, Michal Povazan, Kathrin Reetz, Sandro Romanzetti, Thomas Klockgether, Jennifer Faber

**Affiliations:** a German Center for Neurodegenerative Diseases (DZNE), Bonn, Germany; b A.A. Martinos Center for Biomedical Imaging, Massachusetts General Hospital, Boston, MA, U.S.; c Department of Radiology, Harvard Medical School, Boston, MA, U.S.; d Department of Neuroradiology, University Hospital Bonn, Germany; e Department of Neurology and Center for Translational Neuro- and Behavioral Sciences, University Hospital Essen, University of Duisburg-Essen, Germany; f Ataxia Centre, Department of Clinical and Movement Neurosciences, UCL Queen Square Institute of Neurology, London, UK; g National Hospital for Neurology and Neurosurgery, University College London Hospitals NHS Foundation Trust, London, UK; h Department of Neurology, Donders Institute for Brain, Cognition, and Behaviour, Radboud university medical center, Nijmegen, The Netherlands; i Neurology department, Rijnstate Hospital, Arnhem, the Netherlands; j Department of Neurology, University of Groningen, University Medical Center Groningen, Groningen, the Netherlands; k Johns Hopkins University School of Medicine, Baltimore, MD, U.S.; l Department of Neurology, University Hospital Heidelberg, Heidelberg, Germany; m Center for Magnetic Resonance Research, Department of Radiology, University of Minnesota, Minneapolis, MN, USA; n Department of Neurology, RWTH Aachen University, Germany; o JARA-Brain Institute Molecular Neuroscience and Neuroimaging, Forschungszentrum Jülich, Germany; p Department of Neurology, University Hospital Bonn, Germany

**Keywords:** Ataxia, atrophy, cerebellum, z-scores

## Abstract

**Background:**

Cerebellar atrophy is the neuropathological hallmark of most ataxias. Hence, quantifying the volume of the cerebellar grey and white matter is of great interest. In this study, we aim to identify volume differences in the cerebellum between spinocerebellar ataxia type 1 (SCA1), SCA3 and SCA6 as well as multiple system atrophy of cerebellar type (MSA-C).

**Methods:**

Our cross-sectional data set comprised mutation carriers of SCA1 (N=12), SCA3 (N=62), SCA6 (N=14), as well as MSA-C patients (N=16). Cerebellar volumes were obtained from T1-weighted magnetic resonance images. To compare the different atrophy patterns, we performed a z-transformation and plotted the intercept of each patient group’s model at the mean of 7 years of ataxia duration as well as at the mean ataxia severity of 14 points in the SARA sum score. In addition, we plotted the extrapolation at ataxia duration of 0 years as well as 0 points in the SARA sum score.

**Results:**

Patients with MSA-C demonstrated the most pronounced volume loss, particularly in the cerebellar white matter, at the late time intercept. Patients with SCA6 showed a pronounced volume loss in cerebellar grey matter with increasing ataxia severity compared to all other patient groups. MSA-C, SCA1 and SCA3 showed a prominent atrophy of the cerebellar white matter.

**Conclusion:**

Our results (i) confirmed SCA6 being considered as a pure cerebellar gray matter disease, (ii) emphasise the involvement of cerebellar white matter in the neurophatology of SCA1, SCA3 and MSA-C, and (iii) reflect the rapid clinical progression in MSA-C.

## 1. Introduction

Among the adult-onset degenerative cerebellar ataxias, the autosomal dominantly inherited polyglutamine spinocerebellar ataxias type 1 (SCA1), SCA3, and SCA6, and the sporadic multiple system atrophy of cerebellar type (MSA-C) are most common [1]. SCA1, SCA3 and SCA6 are caused by translated CAG repeat expansion mutations of variable length in the respective genes. The mutations result in formation of abnormal disease proteins containing elongated polyglutamine stretches [1]. MSA-C is neuropathologically defined by the presence of alpha-synuclein-positive inclusions in oligodendroglia. The diagnosis of MSA-C relies on clinical features, particularly including severe autonomic failure [2, 3]. SCA1, SCA3, and MSA-C are multisystemic diseases that not only affect the cerebellum, but also involve the spinal cord, brainstem, basal ganglia, and other regions of the central nervous system. Ataxia is the primary clinical feature, with additional other non-ataxia symptoms such as spasticity, rigidity or ophthalmoparesis. In contrast, SCA6 is considered as an almost purely cerebellar disease characterised by isolated cerebellar ataxia without major non-ataxia signs [1, 4]. Previous neuropathological and MRI studies examining the atrophy patterns of SCA1, SCA3, SCA6, and MSA-C have, to date, predominantly used voxel-based morphometry and emphasized the involvement of extracerebellar structures [5–9]. While voxel-wise analysis can be useful for detecting subtle or widespread changes in brain structure, it is essential to focus on specific regions of the cerebellum when studying cerebellar ataxias. Recent progress in the MRI morphometric analysis of the cerebellum allows detailed quantitative assessment of the cerebellum at the lobular level and precise delineation of cerebellar white matter [10].

The aim of this study was the investigation of the pattern of cerebellar grey and white matter atrophy in SCA1, SCA3, SCA6 and MSA-C. We applied a z-transformation to correct for age effects and used a linear model with read-outs at defined time points of the disease course and degrees of ataxia severity, to allow the comparison of the amount and pattern of cerebellar atrophy between SCA1, SCA3, SCA6 and MSA-C.

## 2. Methods

### 2.1. Participants

Cross-sectional data of SCA1 (*N* = 12), SCA3 (*N* = 62), SCA6 (*N* = 14) and MSA-C (*N* = 16) were analysed. In addition, healthy controls (HCs) (*N* = 292) were analysed and used as a reference for the z-transformation. All subjects participated in ongoing observational studies (ESMI, SCA Registry, DANCER, DELCODE) at 8 European and 2 US sites, and were enrolled between 2017 and 2022. All participants gave their written informed consent according to the declaration of Helsinki. SCA diagnosis was confirmed by diagnostic genetic testing, MSA-C was diagnosed applying the diagnostic criteria from 2008 [2]. The new diagnostic criteria of Wenning et al. [3] could not be applied retrospectively. Ataxia severity was assessed using the Scale of Assessment and Rating of Ataxia (SARA) [11]. We only included ataxic patients with SARA sum scores higher or equal than the established cut-off value of 3 [11, 12]. Ataxia onset was defined as the first occurrence of gait disturbances, and the reported ataxia duration in years was calculated accordingly.

### 2.2. Imaging protocol

All participants were scanned on 3T SIEMENS scanners, with a 32-channel head coil, using a standardised T1-weighted magnetisation-prepared rapid gradient-echo (MPRAGE) sequence. Sequence parameters were as follows: repetition time (TR) = 2500 ms, echo time (TE) = 4.37 ms, inversion time (TI) = 1100 ms, flip angle = 7°, field of view 256 × 256 mm^2^ and 192 slices, voxel size = 1mm isotropic.

### 2.3. Image analysis

We used *CerebNet* [10] for the automated sub-segmentation of the cerebellum into the following volumes (for hemispheric volumes, each volume corresponds to the combined volumes of left and right hemisphere): anterior lobe (consisting of the lobules I-V), superior posterior lobe (consisting of the lobules VI-VII), inferior posterior lobe (consisting of the lobules VIII-IX) and the flocculonodular lobe (corresponding to lobule X) and the midline vermis as well as the cerebellar white matter (cWM). In addition we analysed the combined volume of cerebellar grey matter (cGM) comprised of all hemispheric volumes plus vermis. All scans were visually inspected.

**Figure 1:**
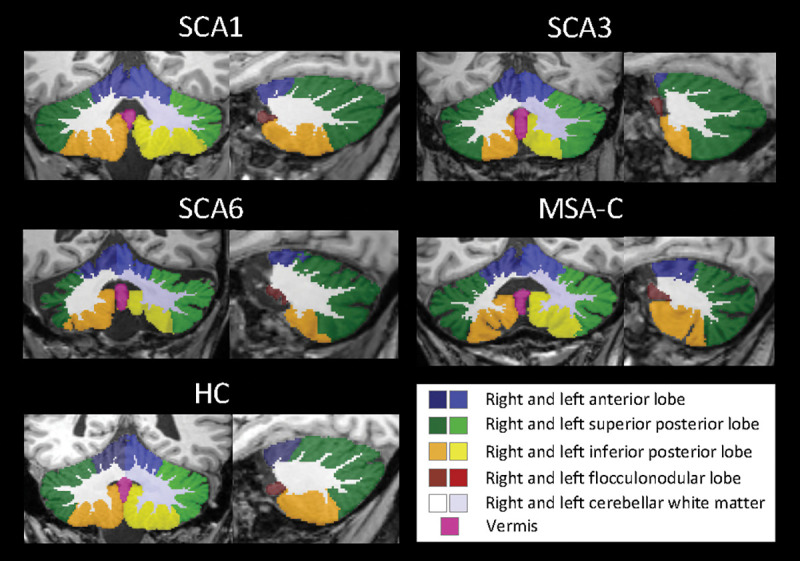
Segmentation examples of a fully automated cerebellar segmentation in a SCA1, SCA3, SCA6 and MSA-C patients as well as in a HC, projected onto a coronal and sagittal slice.

Exemplary cases of the automated cerebellar segmentations are shown in Figure 1.

To account for individual differences in head size, we used estimated total intracranial volume (eTIV) calculated with *FreeSurfer* (version 6.0.0) [13]. Normalised cerebellar volumes were calculated by dividing the raw cerebellar volumes by the individual’s eTIV.

### 2.4. Statistical analysis of cerebellar atrophy in relation to ataxia duration and ataxia severity

All statistical analyses were performed using R Software for Statistical Computing, version 4.2.3 [14]. In order to compare volumes across the different diseases, we z-transformed each normalised volume in relation to HCs to compensate for age-related atrophy, as described previously [15]. Here, a z-score of 0 corresponds to the respective, expected mean in HCs of the same age. A z-score of ±1, ±2 etc. corresponds to ±1, ±2 etc. standard deviations below or above the expected mean in HCs of the same age [15]. The resulting z-scores were used for all subsequent statistical analyses.

In order to compare the different ataxias at the same time points of ataxia duration and degrees of ataxia severity the following stepwise approach was performed for each considered cerebellar volume. First, we plotted the z-scores for each cerebellar volume against the reported ataxia duration in years as well as ataxia severity assessed with the SARA sum score. Here, we applied a linear model for each disease and calculated the coefficient of determination (*R*^2^) and p-value for every correlation. Moreover, quadratic and cubic models were also established. Second, we defined the points for comparison: (i) we calculated the overall mean ataxia duration and ataxia severity for all patient groups, which were 7 years of ataxia duration and a SARA sum score of 14, and (ii) we used the extrapolation at 0 years of ataxia duration and the absence of ataxia (SARA sum score of 0). Accordingly, the intercept volume z-score of the linear interpolation line at 0 years and 7 years of ataxia duration, and at a SARA sum score of 0 and 14, were extracted for each disease. Finally, radar plots of these intercept z-scores for each considered volume were used to visualise the degree and patterns of cerebellar atrophy in SCA1, SCA3, SCA6 and MSA-C at 0 and 7 years of disease duration as well as an ataxia severity of 0 and 14 points in the SARA sum score.

## 3. Results

Demographic and characterising data are summarised in Table 1. SCA1 had the earliest age of onset, while SCA3 patients the longest reported ataxia duration, followed by SCA6. Notably, MSA-C was associated with the highest SARA sum scores among all studied ataxia groups.

### 3.1. Cerebellar atrophy in relation to ataxia duration and ataxia severity

The linear model of volume z-scores and ataxia duration as well as severity are given in Figure 2 and Figure 3. The coefficient of determination (*R*^2^) as well as the p-value representing evidence of a linear relationship are given in the superior left corner of each subplot. Using quadratic and cubic models, neither the residual versus fitted values plots nor the Q-Q plots showed substantial improvement in comparison to the linear model. Thus, given the limited sample size we based the further analyses on the linear model (Supplement Figures 4– 15).

**Figure 2:**
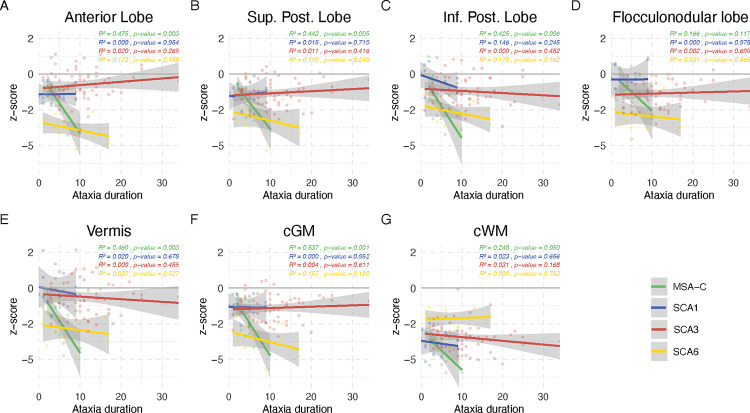
Relation of cerebellar volumes and ataxia duration in years The z-scores of each cerebellar sub-segmented region are plotted against the reported ataxia duration in years for SCA1 (in blue), SCA3 (in red), SCA6 (in yellow) and MSA-C (in green). Linear interpolation was applied with the 95% confidence intervals given as shaded grey areas. Values of the coefficient of determination (*R*^2^) as well as the p-value are given for each disease, respectively.

The relation of cerebellar volumes and ataxia duration in years is shown in Figure 2. Overall, most volumes decreased with increased ataxia duration. The steepest decline of all cerebellar grey and white matter volumes was observed in MSA-C, followed by SCA6 except for cerebellar white matter. In SCA1, the anterior, superior posterior, and flocculonodular lobe, and in SCA3 the anterior, superior posterior and flocculonodular lobe as well as aggregated cGM, showed a not significant slight volume increase with longer ataxia duration.

**Figure 3:**
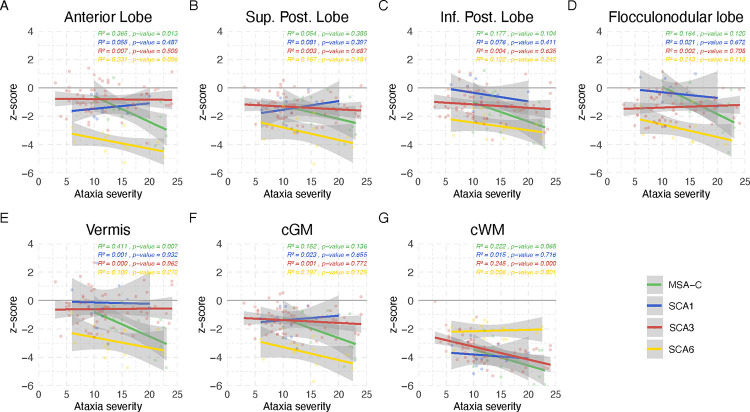
Relation of cerebellar volumes and ataxia severity The z-scores of each cerebellar sub-segmented region are plotted against ataxia severity measured with SARA sum score for SCA1 (in blue), SCA3 (in red), SCA6 (in yellow) and MSA-C (in green). Linear interpolation was applied with the 95% confidence intervals given as shaded grey areas. Values of the coefficient of determination (*R*^2^) as well as the p-value are given for each disease, respectively.

With regard to ataxia severity, most volumes decreased with increased ataxia severity. Overall, MSA-C and SCA6 showed the steepest decline in all grey matter volumes, and MSA-C additionally in the white matter volume, with exception for cWM in SCA6 that presented a slight volume increase. In SCA1 the anterior and superior posterior lobe, as well as cGM, and in SCA3 the flocculonodular lobe and the vermis, as well as in SCA6 the cerebellar white matter showed a not significant slight volume increase with increased ataxia severity. The relation between cerebellar volumes and ataxia severity is shown in Figure 3.

*R*^2^ values were overall relatively small, and ranged between 0 and 0.54. Generally, the highest *R*^2^ was observed in MSA-C followed by SCA6. In MSA-C *R*^2^ was emphasised in relation to ataxia duration with the maximum of *R*^2^ = 0.537 for cGM. In SCA6 *R*^2^ was emphasised in relation to ataxia severity. SCA1 and SCA3 showed very small values of *R*^2^, except for the relation of cWM and ataxia severity in SCA3. In MSA-C the correlation with ataxia duration of all cerebellar volumes reached statistical significance with p ≤ 0.05, except for the flocculonodular lobe. The correlation with ataxia severity reached statistical significance in all volumes, except the anterior lobe and vermis and MSA-C. In the SCAs, only the correlation of cerebellar white matter and ataxia severity in SCA3 reached statistical significance with a p ≤ 0.05.

**Figure 4:**
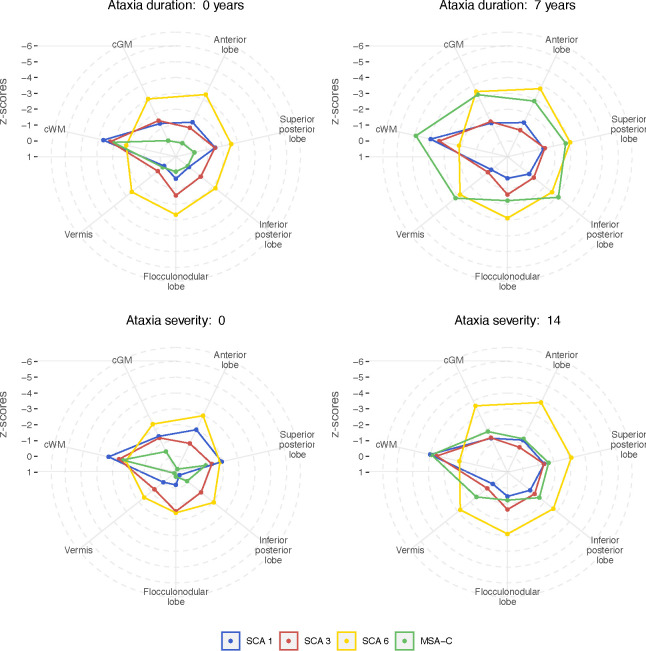
Radar plot of atrophy in SCA1, SCA3, SCA6 and MSA-C at ataxia duration equal to 0 and 7 years and at ataxia severity of a SARA sum score equal to 0 and 14. For the each volume the intercept at an ataxia duration of 0 years (A) as well as the mean ataxia duration of 7 years (B) from the linear interpolation of volume z-scores against ataxia duration, Figure 2, is given. Similarly, the intercept at SARA sum score of 0 (C) as well as the mean SARA sum score of 14 (D) from the linear interpolation of volume z-scores against ataxia severity, Figure 3, is given. A z-score of 0 corresponds to the mean of healthy control distribution, while a z-score of ±1, ±2 etc. correspond to values which are 1, 2 etc. standard deviations (SD) of the distributions in HC above or below the mean in HC, respectively.

To visualise for each disease the pattern and extent of atrophy at certain time points of ataxia duration and degrees of ataxia severity, we displayed the linear interpolated z-scores for the volumes of the anterior, superior posterior and inferior posterior and flocculonodular lobe and vermis as well as cGM and cWM in radar plots. Figure 4 shows radar plots of the extrapolation at the time of ataxia onset (ataxia duration of 0 years) and at 7 years after ataxia onset, as well as for the intercept at SARA sum scores of 0 and 14. Comparison of the radar plots at ataxia onset and at 7 years after onset revealed a strong atrophy increase in MSA-C, whereas the increase was less pronounced in the SCAs. Comparison of the plots at SARA sum score of 0 and of 14 revealed an emphasized atrophy increase in SCA6 and MSA-C, whereas the increase was only minor in SCA1 and SCA3. Moreover, in SCA1, SCA3 and MSA-C cWM volume was consistently affected, in contrast to SCA6, where the cWM was considerably less affected than the grey matter structures.

## 4. Discussion

We used a z-transformation to study the degree and distribution of cerebellar atrophy in SCA1, SCA3, SCA6 and MSA-C at certain levels of ataxia duration and ataxia severity, while accounting for the healthy aging effect. We could demonstrate a strong coherence between ataxia severity and grey matter atrophy in SCA6, underlining the consideration of SCA6 as a pure cerebellar disease. In contrast, volume loss of the cerebellar white matter was prominent in MSA-C, SCA1 and SCA3. Overall MSA-C showed the steepest decline of all cerebellar white and grey matter volumes in particular in relation to ataxia duration.

Since ataxias are rare diseases, patient populations are often small and additionally, as in our sample, differ in duration and severity of ataxia, making comparison between entities difficult. We chose the applied approach to overcome this limitation and used radar plots to visualise the differences in atrophy patterns between diseases at certain levels of ataxia duration and severity.

Our results are in line with previous studies mainly of voxel-based-morphometry as well as volumetry in SCAs and MSA-C [5, 6, 8, 16]. Notably, throughout the different considered intersections of ataxia duration and ataxia severity, we confirmed earlier findings that, in contrast to other SCA genotypes, atrophy in SCA6 was mainly restricted to the cerebellar GM almost excluding the WM [17, 18]. While cerebellar GM atrophy at the extrapolated point of 0 SARA sum score has no clear discernible pattern across the diseases, it becomes markedly emphasised in SCA6 with increasing ataxia severity in comparison to SCA1, SCA3 and MSA-C. Thus, in SCA6 cerebellar atrophy seems to account almost solely for the clinically observed ataxia. In contrast, it is known, that the neuropathology in the other, multi-systemic disorders SCA1, SCA3 and MSA-C also involves other parts of the central nervous system, e.g. brainstem and basal ganglia [1, 15, 19]. Here, the resulting non-ataxia symptoms, e.g., spasticity or rigidity, probably exacerbate motor and coordination impairments. In other words, by concept, the impairments measured by the SARA scale primarily capture ataxia but may be aggravated by other non-ataxia symptoms. With regard to ataxia duration, MSA-C shows the most pronounced cerebellar grey and white matter atrophy with increasing ataxia duration. This observation reflects the known rapid clinical progression in MSA-C [20]. For the clinical diagnosis of MSA, qualitative imaging features have recently been included as mandatory features [3], underlining their importance. In this analysis, due to the recruitment period, only the former clinical criteria [2] found the basis for the clinical diagnosis of probable MSA-C. We excluded one patient who met these former clinical diagnostic criteria but had an unexpectedly long disease duration of > 30 years. Future studies might be more specific with potentially improved diagnostic accuracy due to the revised clinical criteria. Cerebellar white matter atrophy was pronounced in MSA-C, SCA1 and SCA3, with the steepest and significant decline relative to ataxia severity in SCA3 and MSA-C. In MSA-C, alpha-synuclein deposition in oligodendrocytes represents the central neuropathological changes. However, also in SCA3 there is increasing evidence for a strong involvement of oligodendrocytes in the disease-specific neuropathology [21].

Our approach allowed the comparison of the different diseases at specific levels of duration and severity of ataxia, yet these results are based on indirect measurements and are therefore subject to limitations that must be considered. The number of available participants, along with their relatively short spans of ataxia duration and severity levels, obviously can impact the linear model. Within the SCAs, in particular SCA1 and SCA3, we found in a minority of volumes a contra-intuitive increase of volumes with increasing ataxia duration or severity. We hypothesise that this is either due to noise or a selection bias with a biased focus on the proportion of less affected patients in advanced stages. Severely affected patients suffer substantial restrictions in mobility and are often no longer able to travel to study visits. Thus, the complete spectrum of advanced stage patients might not be represented, but rather, there may be a positive-selection of those who are still reasonably mobile despite an advanced SARA score. Studies with larger sample sizes and in addition ideally longitudinal data are needed to study the differences between diseases more reliably at comparable levels of ataxia severity and duration. Despite the aforementioned limitations, our results are able to illustrate main principles using comparative visualisations of volumetric changes in cerebellar grey and white matter in MSA-C, SCA1, SCA3 and SCA6 and thereby may inform further studies on a broader data basis.

## 5. Conclusion

Application of z-transformation to correct for age effects and the use of a linear model to read-out volumetric values at distinct levels of disease duration and ataxia severity allowed comparison of the extend and pattern of cerebellar atrophy between SCA1, SCA3, SCA6 and MSA-C. In summary, our results confirmed that SCA6 is primarily a pure cerebellar disease with markedly emphasised cGM atrophy. In contrast, a prominent involvement of cWM was found in SCA1, SCA3 and MSA-C. The well-known rapid clinical progression in MSA-C was also reflected in impressive volume loss later in the disease course. Further studies in larger longitudinal samples are needed to confirm our findings and put them on a broader basis.

## Figures and Tables

**Table 1:** Demographic and cohort characteristics

Group entity	Number (Female/ Male)	Age at scan^1^ mean (SD)	Ataxia duration mean (SD)	SARA sum score mean (SD)	CAG repeats^2^ mean (SD)

SCA1	12 (7/5)	45.1 (9.6)	3.9 (3.3)	12.1 (3.9)	48.3 (4.2)
SCA3	62 (25/37)	50.7 (10.6)	10.8 (6.5)	12.8 (6.2)	67.8 (8.0)
SCA6	14 (4/10)	64.2 (8.6)	7.8 (5.5)	13.3 (4.9)	21.8 (0.5)
MSA-C	16 (6/10)	64.5 (8.2)	4.4 (2.1)	17.2 (5.2)	n.a.
HC	292 (160/132)	63.5 (12.6)	n.a.	n.a.	n.a.

SD - standard deviation; SCA - Spinocerebellar ataxia; MSA-C - Multiple system atrophy of cerebellar type; HC - Healthy control; n.a., not applicable

1 in years

2 of the longer allele

## Data Availability

This declaration is not applicable.

## References

[1] Klockgether T., Mariotti C., Paulson H., Spinocerebellar ataxia, Nature reviews. Disease primers 5 (1). doi:10.1038/s41572-019-0074-3. 30975995

[2] Gilman S., Wenning G., Low P., Brooks D., Mathias C., Trojanowski J., Wood N., Colosimo C., Dürr A., Fowler C., Kaufmann H., Klockgether T., Lees A., Poewe W., Quinn N., Revesz T., Robertson D., Sandroni P., Seppi K., Vidailhet M., Second consensus statement on the diagnosis of multiple system atrophy, Neurology 71 (9) (2008) 670–6. doi:10.1212/01.wnl.0000324625.00404.15. 18725592 PMC2676993

[3] Wenning G., Stankovic I., Vignatelli L., Fanciulli A., Calandra-Buonaura G., Seppi K., Palma J., Meissner W., Krismer F., Berg D., Cortelli P., Freeman R., Halliday G., Höglinger G., Lang A., Ling H., Litvan I., Low P., Miki Y., Panicker J., Pellecchia M., Quinn N., Sakakibara R., Stamelou M., Tolosa E., Tsuji S., Warner T., Poewe W., Kaufmann H., Rhe movement disorder society criteria for the diagnosis of multiple system atrophy, Movement Disorders 37 (6) (2022) 1131–1148. doi:10.1002/mds.29005. 35445419 PMC9321158

[4] Rentiya Z., Hutnik R., Mekkam Y., Bae J., The pathophysiology and clinical manifestations of spinocerebellar ataxia type 6, Cerebellum 19 (3) (2020) 459–464. doi:10.1007/s12311-020-01120-y. 32125675

[5] Hernandez-Castillo C. R., King M., Diedrichsen J., Fernandez-Ruiz J., Unique degeneration signatures in the cerebellar cortex for spinocerebellar ataxias 2, 3, and 7, NeuroImage: Clinical 20 (2018) 931–938. doi:10.1016/j.nicl.2018.09.026. 30308379 PMC6178193

[6] Reetz K., Costa A. S., Mirzazade S., Lehmann A., Juzek A., Rakowicz M., Boguslawska R., Schols L., Linnemann C., Mariotti C., Grisoli M., Durr A., van de Warrenburg B. P., Timmann D., Pandolfo M., Bauer P., Jacobi H., Hauser T. K., Klockgether T., Schulz J. B., axia Study Group I., Genotype-specific patterns of atrophy progression are more sensitive than clinical decline in sca1, sca3 and sca6, Brain 136 (Pt 3) (2013) 905–17. doi:10.1093/brain/aws369. 23423669

[7] Deistung A., Jäschke D., Draganova R., Pfaffenrot V., Hulst T., Steiner K. M., Thieme A., Giordano I. A., Klockgether T., Tunc S., Münchau A., Minnerop M., Göricke S. L., Reichenbach J. R., Timmann D., Quantitative susceptibility mapping reveals alterations of dentate nuclei in common types of degenerative cerebellar ataxias, Brain Commun 4 (1) (2022) fcab306. doi:10.1093/braincomms/fcab306. 35291442 PMC8914888

[8] Adanyeguh I. M., Perlbarg V., Henry P.-G., Rinaldi D., Petit E., Valabregue R., Brice A., Durr A., Mochel F., Autosomal dominant cerebellar ataxias: Imaging biomarkers with high effect sizes, NeuroImage: Clinical 19 (2018) 858–867. doi:10.1016/j.nicl.2018.06.011. 29922574 PMC6005808

[9] Chandrasekaran J., Petit E., Park Y. W., du Montcel S. T., Joers J. M., Deelchand D. K., Povazan M., Banan G., Valabregue R., Ehses P., Faber J., Coupe P., Onyike C. U., Barker P. B., Schmahmann J. D., Ratai E. M., Subramony S. H., Mareci T. H., Bushara K. O., Paulson H., Durr A., Klockgether T., Ashizawa T., Lenglet C., Oz G., Consortium R., Clinically meaningful magnetic resonance endpoints sensitive to preataxic spinocerebellar ataxia types 1 and 3, Ann Neuroldoi:10.1002/ana.26573. PMC10261544 36511514

[10] Faber J., Kügler D., Bahrami E., Heinz L., Timmann D., Ernst T., Deike-Hofmann K., Klockgether T., van de Warrenburg B., van Gaalen J., Reetz K., Romanzetti S., Oz G., Joers J., Diedrichsen J., Group E. M. S., Reuter M., Cerebnet: A fast and reliable deep-learning pipeline for detailed cerebellum sub-segmentation, Neuroimage 264 (2022) 119703. doi:10.1016/j.neuroimage.2022.119703. 36349595 PMC9771831

[11] Schmitz-Hübsch T., du Montcel S. T., Baliko L., Berciano J., Boesch S., Depondt C., Giunti P., Globas C., Infante J., Kang J. S., Kremer B., Mariotti C., Melegh B., Pandolfo M., Rakowicz M., Ribai P., Rola R., Schöls L., Szymanski S., van de Warrenburg B. P., Dürr A., Klockgether T., Scale for the assessment and rating of ataxia: development of a new clinical scale, Neurology 66 (11) (2006) 1717–1720. 16769946 10.1212/01.wnl.0000219042.60538.92

[12] Maas R. P., van Gaalen J., Klockgether T., van de Warrenburg B. P., The preclinical stage of spinocerebellar ataxias, Neurology 85 (1) (2015) 96–103. doi:10.1212/WNL.0000000000001711. 26062625

[13] Buckner R. L., Head D., Parker J., Fotenos A. F., Marcus D., Morris J. C., Snyder A. Z., A unified approach for morphometric and functional data analysis in young, old, and demented adults using automated atlas-based head size normalization: reliability and validation against manual measurement of total intracranial volume, Neuroimage 23 (2) (2004) 724–38. doi:10.1016/j.neuroimage.2004.06.018. 15488422

[14] R Core Team, R: A Language and Environment for Statistical Computing, R Foundation for Statistical Computing, Vienna, Austria (2023).

[15] Faber J., Schaprian T., Berkan K., Reetz K., França M. C. Jr, de Rezende T. J. R., Hong J., Liao W., van de Warrenburg B., van Gaalen J., Durr A., Mochel F., Giunti P., Garcia-Moreno H., Schoels L., Hengel H., Synofzik M., Bender B., Oz G., Joers J., de Vries J. J., Kang J.-S., Timmann-Braun D., Jacobi H., Infante J., Joules R., Romanzetti S., Diedrichsen J., Schmid M., Wolz R., Klockgether T., Regional brain and spinal cord volume loss in spinocerebellar ataxia type 3, Movement Disordersdoi:10.1002/mds.28610. PMC9521507 33951232

[16] Rezende T. J. R., de Paiva J. L. R., Martinez A. R. M., Lopes-Cendes I., Pedroso J. L., Barsottini O. G. P., Cendes F., França M. C. Jr, Structural signature of sca3: From presymptomatic to late disease stages, Annals of Neurology 84 (3) (2018) 401–408. doi:10.1002/ana.25297. 30014526

[17] Schulz J. B., Borkert J., Wolf S., Schmitz-Hübsch T., Rakowicz M., Mariotti C., Schoels L., Timmann D., van de Warrenburg B., Dürr A., Pandolfo M., Kang J.-S., Mandly A. G., Nägele T., Grisoli M., Boguslawska R., Bauer P., Klockgether T., Hauser T.-K., Visualization, quantification and correlation of brain atrophy with clinical symptoms in spinocerebellar ataxia types 1, 3 and 6, NeuroImage 49 (1) (2010) 158–168. doi:10.1016/j.neuroimage.2009.07.027. 19631275

[18] Lukas C., Schöls L., Bellenberg B., Rüb U., Przuntek H., Schmid G., Köster O., Suchan B., Dissociation of grey and white matter reduction in spinocerebellar ataxia type 3 and 6: a voxel-based morphometry study, Neurosci Lett 408 (3) (2006) 230–235, epub 2006 Sep 26. doi:10.1016/j.neulet.2006.09.007. 17005321

[19] Faber J., Giordano I., Jiang X., Kindler C., Spottke A., Acosta-Cabronero J., Nestor P. J., Machts J., Düzel E., Vielhaber S., Speck O., Dudesek A., Kamm C., Scheef L., Klockgether T., Prominent white matter involvement in multiple system atrophy of cerebellar type, Movement Disorders 35 (5) (2020) 816–824. doi:10.1002/mds.27987. 31994808

[20] Oender D., Faber J., Wilke C., Schaprian T., Lakghomi A., Mengel D., Schöls L., Traschütz A., Fleszar Z., Dufke C., Vielhaber S., Machts J., Giordano I., Grobe-Einsler M., Klopstock T., Stendel C., Boesch S., Nachbauer W., Timmann-Braun D., Thieme A., Kamm C., Dudesek A., Tallaksen C., Wedding I., Filla A., Schmid M., Synofzik M., Klockgether T., Evolution of clinical outcome measures and biomarkers in sporadic adult-onset degenerative ataxia, Movement Disorders 38 (4) (2023) 1654–664. doi:10.1002/mds.29324. 36695111

[21] Schuster K. H., Zalon A. J., Zhang H., DiFranco D. M., Stec N. R., Haque Z., Blumenstein K. G., Pierce A. M., Guan Y., Paulson H. L., McLoughlin H. S., Impaired oligodendrocyte maturation is an early feature in sca3 disease pathogenesis, J Neurosci 42 (8) (2022) 1604–1617. doi:10.1523/JNEUROSCI.1954-20.2021. 35042771 PMC8883861

